# Understanding the *Why*: Patient, Parent, and Oncologist Perspectives on Prognostic Communication Preferences in Advanced Childhood Cancer

**DOI:** 10.3390/children12091140

**Published:** 2025-08-28

**Authors:** Erin Batchelor, Rachel A. Kentor, Calliope Reeves, Harmony Farner, Shoshana Mehler, Caroline Christianson, Erica C. Kaye

**Affiliations:** 1 Department of Oncology, St Jude Children’s Research Hospital, Memphis, TN 38105, USA; erin.batchelor@stjude.org (E.B.); reecm-26@rhodes.edu (C.R.); harmony.farner@stjude.org (H.F.); shoshana.mehler@stjude.org (S.M.); 2 Divisions of Palliative Care and Psychology, Department of Pediatrics, Baylor College of Medicine, Texas Children’s Hospital, Houston, TX 77030, USA; rxkentor@texaschildrens.org; 3 Rhodes College, Memphis, TN 38112, USA; 4 Division of Pediatric Hematology/Oncology, NYU Langone Health, New York, NY 10016, USA; caroline.christianson@nyulangone.org

**Keywords:** pediatric, cancer, communication, prognosis, adolescents and young adults, parents, caregivers

## Abstract

**Highlights:**

**What are the main findings?**

- Patients, parents, and oncologists identified four distinct themes of factors that influenced their prognostic communication preferences.

**What is the implication of the main finding?**

- These findings offer a novel approach for eliciting and honoring preferences for prognostic communication in the setting of advanced childhood cancer.
- These findings highlight the need to conduct further research to develop strategies for eliciting prognostic communication preferences and the reasons behind them, as well as honoring these preferences in practice.

**Abstract:**

Background/Objectives: High-quality prognostic communication in pediatric oncology is essential to support informed decision making, foster trust, and honor goals of care. While families differ in their preferences for how prognosis is discussed, clinicians often rely on assumptions rather than directly eliciting these preferences, risking misalignment and distress. To address this gap, we aimed to characterize patient, parent, and oncologist perspectives on key variables, experiences, and circumstances that influence their preferences for prognostic communication. Methods: Semi-structured interviews were conducted with 85 participants (25 patients aged 12–25, 40 parents, and 20 oncologists) to elicit their preferences for prognostic communication and the reasons behind these preferences. Rapid analysis was conducted to generate themes and identify patterns and variances across participant cohorts. Results: Four distinct themes underpinning prognostic communication preferences were described by patients, parents, and oncologists: (1) personality, ideals, and values; (2) general life experiences; (3) prior interactions with the medical system; and (4) faith. Participants emphasized that personal identity and prior experiences significantly shaped how they wished to receive prognostic information. Clinicians and parents often linked preferences to core aspects of their professional or caregiver identities. Conclusions: Understanding the individualized factors shaping communication preferences can guide clinicians in tailoring prognostic discussions. Open-ended inquiry into identity, personal values, and past experiences can foster respectful, personalized communication in pediatric oncology. Further research is needed to determine best practices for prompting discussion about prognostic communication preferences that incorporates the reasons underpinning these preferences.

## 1. Introduction

How oncologists talk about prognosis significantly influences how pediatric patients and their parents understand their disease, affecting their ability to make informed decisions [1,2]. In the context of treatment-refractory disease, parents of children with advanced cancer are more likely to report a goal of comfort when they recall their child’s oncologist sharing information about prognosis [3]. Beyond influencing goals of care, high-quality prognostic communication also strengthens the parent–oncologist relationship and trust in the physician, reduces decisional regret, and supports parental hope [2,4,5,6]. Central to these outcomes is the process of shared decision making (SDM), in which clinicians and families work together to align medical recommendations with the child’s and family’s values, goals, and preferences [4]. Effective prognostic disclosure lays the foundation for SDM by fostering mutual understanding and emotional readiness to engage in complex choices.

Recent evidence demonstrates that pediatric patients with advanced cancer, their parents, and oncologists have diverse preferences and recommendations for prognostic disclosure, underscoring the importance of tailoring communication to align with individual needs [7]. When clinicians acknowledge and honor a family’s communication preferences, parents report feeling a greater sense of control and a belief that their team has done everything possible for their child [8,9]. Yet data suggest that pediatric oncologists often infer what prognostic information families want to hear, and withhold information at certain timepoints in an effort to preserve therapeutic alliance or mitigate distress [10,11]. Given the broad range of preferences endorsed by different patients and parents, this inference risks misalignment with a given family’s preference and may be subject to implicit bias [12].

To mitigate this concern, patient- and parent-driven strategies are needed to guide clinicians in eliciting prognostic communication preferences prior to difficult conversations [13]. Skillful elicitation of preferences relies on awareness and sensitivity to the factors that shape individualized communication preferences, which remain underexplored. Gaining insight into the reasons underpinning communication preferences can help clinicians tailor their provision of prognostic information. Similarly, understanding common themes that influence communication preferences can help clinicians broach conversations to ask about individualized preferences, normalizing the various reasons why people may prefer one approach over another.

To address this gap, we interviewed patients, parents, and pediatric oncologists about the reasons underlying their individual preferences for prognostic communication in the setting of advanced cancer. This paper presents findings from a targeted analysis of the factors identified by patients, parents, and oncologists as instrumental in shaping their communication preferences for prognostic disclosure.

## 2. Materials and Methods

The RIGHTime (Revealing Information Genuinely and Honestly across Time) study is a multi-phase, mixed-methods investigation of the ‘right’ way to talk about prognosis in the setting of advanced childhood cancer. Institutional Review Board approval was obtained from St. Jude Children’s Research Hospital. Study procedures and findings from semi-structured interviews conducted with patients, parents, and oncologists from the first phase of the study are presented here, in accordance with the COREQ (Consolidated Criteria for Reporting Qualitative Research) checklist (Supplemental Table S1) [14].

### 2.1. Eligibility

Three groups were recruited for this study: pediatric cancer patients, parents of children with cancer, and pediatric oncologists. Eligible patients were aged 12–25 years, had a cancer diagnosis considered “advanced”, defined as an estimated survival rate of <50% as determined by an oncologist, and were within three months of diagnosis (cohort 1), disease relapse/progression (cohort 2), or phase I/II trial enrollment (cohort 3). The lower age limit of 12 years is consistent with previous pediatric oncology research [15] defining early adolescence and is additionally considered the age at which children can have the capacity to be competent in decision making [16]. The upper age for the current study was defined by the cut-off age for treatment at the study site. Eligible parents were aged ≥18 years, caregivers of a pediatric cancer patient of any age who received a poor-prognosis diagnosis, and recruited from one of the previously mentioned timepoints or during bereavement 6–24 months after the death of their child (cohort 4). Each participating patient and parent had received care at the central study site at least once during the course of the patient’s illness, with many families also receiving care at other institutions. Eligible clinicians were pediatric oncologists currently employed at the central study and five other pediatric cancer programs within different institutions, representing six centers across five states. Research team members reviewed inpatient and outpatient rosters weekly to assess eligibility. Purposeful sampling [17] was conducted to enroll patients with demographic diversity relating to gender, age, race, ethnicity, disease type (patients/parents), and years in clinical practice (oncologists). Eligible patients and parents were approached in person during clinic visits or over the telephone by research staff to obtain written consent as per institutional policy. Patients and parents from cohorts 1–3 were enrolled as dyads if eligible. Research staff contacted oncologists via email and completed verbal consent.

### 2.2. Interview Guide Development and Data Collection

Semi-structured interviews were employed to allow participants to express a wide range of experiences and perspectives in their own words, while minimizing the influence of researcher assumptions that would be inherent in more structured formats such as surveys or fully standardized interviews. Interview guides were developed for each participant group (patient, parent, and oncologist) using the National Cancer Institute (NCI)’s six core functions of patient–clinician communication [18]. The research team (Table 1) collaborated with bereaved parents to review and refine interview guides for content, clarity, and structure. In-depth discussion of interview guide development has been detailed in previous publications [7,19]. Interviews were conducted by research staff in either a private location or via telephone, as determined by participant preference. Interviews were audio-recorded, transcribed, and de-identified. Interviewers maintained field notes to be reviewed by the research team.

The research team engaged in ongoing reflexivity (i.e., critical self-reflection on how team members’ own identities, experiences, and assumptions may influence the research process) throughout the study. We acknowledged how our professional roles and prior experiences in pediatric oncology could shape data collection and interpretation and sought to mitigate these influences by using a semi-structured interview guide, regularly discussing emerging themes as a team, and grounding our analysis in participants’ own words.

### 2.3. Data Analysis

A rapid qualitative analysis (RQA) approach was selected to organize and synthesize interview data, as it enables stratification and systematic comparison across participant groups while supporting the identification of high-level themes and patterns that can directly inform practice [20,21]. This targeted analysis focused on the NCI core communication domains of enabling patient self-management and exchanging information, with the goal of understanding how participants describe factors that affect prognostic communication preferences. The analysis followed a structured, phased approach. Episode summary templates were created by the research team, organized by interview prompt questions from the relevant core communication domains. Analysists (H.F., S.M., E.C.K.) reviewed transcripts independently to assess interview content and generate key concepts within each section of the episode summaries, focusing on patient, parent, and oncologist perceptions of factors influencing their prognostic communication preferences. Initially, multiple analysts created episode summaries for each transcript to support concept generation until >90% consensus (i.e., overlap in identified concepts and labels across coders) was achieved. Analysts then independently created episode summaries for individual transcripts, meeting weekly as a team to iteratively synthesize concepts and identify high-level themes. Concepts and themes from each episode summary were entered into a matrix to enable the research team to compare patterns across participant groups and timepoints. Theme frequencies were stratified by cohort and reported in the following quartiles: few (0–24%), some (25–49%), most (50–74%), nearly all (75–99%), or all (100%). Participant demographic data were summarized descriptively.

## 3. Results

Eighty-five semi-structured interviews were conducted with patients (*n* = 25), parents (*n* = 40), and oncologists (*n* = 20) [21]. Table 2 presents participant demographic data. Briefly, about two-thirds of patients and parents self-identified as female and white, about one-third as Black or biracial, and about one-eighth as Latino/Hispanic. Similarly, about two-thirds of oncologists self-identified as female and white, about one-third as Asian, and one as Latino/Hispanic. Median interview duration was 25 min for patients (19–38), 53 min for parents (19–140), and 47 min for oncologists (26–88).

Participants were asked a variety of questions about their prognostic communication preferences, and after they shared their perspectives, they were asked to reflect on the open-ended prompt, “Do you have thoughts about why you might feel this way or have these specific preferences?” Analysis of these data generated four core themes underpinning the reasons behind prognostic communication preferences across participant cohorts: personal values, lived experience, prior medical encounters, and faith. Table 3 presents theme frequencies.

### 3.1. Personality, Ideals, and Values

Most patients and parents, and some oncologists, suggested that their personality is the biggest shaper of preferences for prognostic communication. One patient summarized this sentiment: “*I reckon it’s the way I am, what I can say.*” *(Patient 307)* A few used words such as “emotional” or “moody” to describe their personality, while others discussed how they preferred to receive explicit information because of their inquisitive, blunt, analytical, or fact-oriented personalities. One parent explained,

*“That’s just how my brain worked. I need to know how we got here. I do ask probably more questions than some people, but I’m also very blunt. I don’t need things sugarcoated for me. I know that my perception and the way my brain works is not the way everyone else’s does.” *

*(Parent 108)*

Most parents and patients said that they like feeling prepared, making plans of action, and knowing all there is to know. A parent commented,

*“I feel like knowing everything, it prepares me. It keeps me on my Ps and Qs about everything. Also, finding out about certain things when it comes to the chance of being that it’s a rare, severe cancer, it educates me, too.” *

*(Parent 307)*

Similarly, a patient remarked, *“I like to know what is going to happen so I can better be ready for it… Personality-wise, I do like to know what’s going to happen, but I can also roll with the punches pretty well.” (Patient 208)* Other patients and parents acknowledged having an anxious personality and how certain modes of communication better ease their worries. One parent discussed preferences for more forward-focused communication that allows for expectation setting and planning:

*“I’m a very anxious person and schedules help bring the anxiety down in a way that I kind of cope with all of this that’s going on… I kind of want to know where’s this leading? Like where are we a month from now, ideally, right?” *

*(Parent 210)*

Some oncologists also reported that their personality shapes their communication preferences. One oncologist commented on his blunt communication style:

*“My wife says sometimes I’m like [a] robot and just say things the way they are. I don’t know if that’s a good thing or makes it bad. I don’t know about that… if you should be that blunt. I’ve always believed in the bluntness.” *

*(ON4)*

Alternatively, another oncologist identified how an empathic personality has shaped her prognostic communication preferences:

*“But by nature, I’m a very empathetic person. I feel that, you know, telling a mom and dad that your child has cancer or the child is not going to make it… you’re giving that person the worst news of their life… I have a very strong empathy towards that. I feel it inside my heart, and I always feel that needs to be delivered in a right kind and compassionate way.” *

*(ON2)*

Of note, a few parents described their communication preferences as rooted in their identity as their child’s advocate. They shared how their preferences revolved around information that would allow them to best support and advocate for their child. One parent commented that her preferences for hearing upsetting information without her child present centered on a desire to shield the child from painful information:

*“[I need] to protect her. It always goes back to protecting her. I have always said that I would rather take on the worry than her… She already has to do enough… So, if I can take any of that away from her, I would rather take on the worry.” *

*(Parent 209)*

While a few parents described protection to mean sheltering their child from emotional distress, others identified the importance of advocating for or against certain medical interventions. For example, one parent identified the importance of engaging in the shared decision-making process:

*“My wife and I have seen at all three hospitals we’ve been at now, we have directly involved ourselves in advocating for certain procedures to be done or withheld, and the doctors have come back to us afterwards and thanked us and told us that we were right…” *

*(Parent 302)*

One oncologist identified a feeling of ethical obligation to perform her role correctly as central to her prognostic communication practices: “*I just think it’s the right thing to do.” (ON3)* Another oncologist added that her preferences for early prognostic disclosure evolved out of her goal to be a ‘good doctor,’ stating:

*“And then there’s also personal experience of how I came to be an oncologist with my family… and what was a good doctor, a good oncologist… and so that definitely shapes me… I have found that because I was honest from the get-go and throughout the journey that [families] were somehow prepared for it.” *

*(ON17)*

### 3.2. Life Experiences

Some parents, a few patients, and nearly all oncologists expressed the profound impact of life experience on their communication preferences. A parent shared that her desire to have as much prognostic information as possible in order to perform her own independent research comes “*… from my, you know, experiences in my life that I’ve gone through, that I’m going through… I also lost my first child… I lost my mom to pancreatic cancer… So I’m used to it” (Parent 202)* An oncologist similarly agreed that preferences for not sharing difficult information over the phone had been shaped by her life experience, commenting, “*… it’s partly because of who I am, my family does not give bad news over the phone. So that was just how I was always raised” (ON5)*

A few oncologists, but no patients or parents, noted the influence that their own culture and/or country of origin played in their preferences for disclosing prognosis. One oncologist said,

*“I always believed in bluntness, maybe in part because of my past experiences. I come from part of the world where politicians lie to people, people lie to each other. And that I’ve seen the consequences of this. People have forced expectations, and they get it crushed.” *

*(ON4)*

Another oncologist remarked on how she adapts her approach based on the culture of patients and families:

*“… it’s very important to try and be respectful of people’s cultures. And the prognostic and prognosis conversations because that obviously plays a role by cultures. It could be, you know, that represents a whole bunch of things across of either religion. So there’s a lot that you need to bring into those conversations. And other people, you need to bring into them, too.” *

*(ON3)*

A few parents and most oncologists acknowledged that professional training and experience have impacted their communication preferences. Of these participants, most commented on their desire for order and control, a proclivity for scientific or critical thinking, and a need to create a clear plan. One parent explained her communication preferences by stating, “*Yeah, I work and I do statistics for my job and my family, my mom and dad were physicians. My dad was a pediatric surgeon, my mom a pediatrician, she was a pulmonary disease specialist, my sister’s a general ped, sister-in-law radiologist.” (Parent 207)* Another parent discussed how his military career impacted his communication preferences:

*“My background is in military aviation. Brief every flight before we step, so everyone knows what we’re going to do, but we know we’re going up and we’re doing a low level, we’re going to go hit the tanker and then we’re going to fly formation. All that information we brief before we step… You have to know what you’re going to do when everything is good and stable and smooth and everyone has a chance to talk about it, what if it… ?” *

*(Parent 204)*

Most oncologists discussed how medical training influenced their preferences for prognostic disclosure. Some shared specific stories of when and how their prognostic communication preferences were shaped, which most often were during fellowship training. One oncologist remembered:

*“In my fellowship, I had attendings that’s been 7 h talking to a family, crying with them, and I said, ‘I’m never going to do that.’ The other [attending] that said, ‘Patient is going to die, I’m going to go to the next room’—I said, I’m never going to do that. So I decided to take the middle road, because I was shaped by my attendings since I was a fellow… My first year [of fellowship] is what basically shaped me on how I talk to families and how I approach families.” *

*(ON1)*

Similarly, another oncologist honed her preferences by observing what not to do:

*“What has helped me over the years is, during my fellowship, my different mentors—I have very keenly observed how each of them delivered this kind of news or had this conversation and I focused more on where they went wrong… like, you know, answering text while having this conversation… We are physicians, we are busy all the time. So a lot of time, the text is about a patient care, but still, it takes away from what you’re doing for the family at that time.” *

*(ON2)*

### 3.3. Prior Interactions with the Medical System

Some patients, parents, and oncologists described how prior experiences with the health care system impacted their preferences for prognostic communication. Events such as experiencing prior illness or loss, negative encounters with clinicians, or active stressful clinical situations all shaped their preferences. A few parents and some oncologists discussed instances of facing hardships from illness affecting themselves or their family. One parent reflected on how her first child’s death shaped the way she later viewed her preference for detailed prognostic information from the doctor.

*“Having experienced loss [of a child] before, certain things that I know now, if I knew it back then… You know, like with the doctors being vague and not telling me everything and kind of, I wasn’t equipped to make the best decision. So, I asked a lot of questions. I know what’s at stake.” *

*(Parent 205)*

Another parent explained how the illness and death of her grandmother affected her preferences. She commented, “*I like to have control of the situation, so I want to know exactly what we get into. ’Cause my grandma, she died of cancer… They weren’t really telling me if she was going to be okay, or how long she was going to take treatments and all that. I feel like for my son, I want to know all of it, so I can mentally prepare myself.” (Parent 103)*

An oncologist also recounted her experience sitting with her own family member in the ICU and how that helped to shape her communication preferences:

*“My goddaughter… was diagnosed with AML during my training, and then she nearly died when she relapsed in her brain, and it was like a very… I’ve had many nights on an ICU floor and [it has] given me great empathy for families because I can say I’ve been there.” *

*(ON16)*

A few patients and parents, but no oncologists, discussed how prior negative medical encounters affected their communication preferences. One patient cited suboptimal information exchange in the past as the reason for his current preferences, stating that his desire for clear, direct prognostic disclosure now is “*because we’ve had such poor communication in the past.” (Patient 304)* Parental preferences were also shaped by previous negative encounters with the health care system. One parent stated, *“So once I had that experience with that doctor… I knew now to, like, get ahead of the doctors and teach them what I needed before they opened their mouth.” (Parent 410)* Another parent described an experience where her child’s cancer symptoms were repeatedly disregarded, stating: “*They didn’t want to do blood work. And I insisted and insisted, ‘hey, I’m not leaving this office tonight until you do it.’ And then, the truth is revealed. ‘Oh my God, you got to go to the hospital right away.’” (Parent 205)*

A few patients and parents indicated how recurrent or current experiences with childhood cancer also shaped their prognostic communication preferences. Of these participants, most discussed the difficulty of the childhood cancer journey, and how this experience transformed the way they prefer to hear prognosis. One patient stated: “*I have experience from my first round, like when they told me all the information and when they didn’t, it helped when I did know all of it. So, that’s one of the reasons I feel like I want to know all of it.” (Patient 305)*

One parent described how earlier communication experiences during her child’s treatment shaped her later preference for gently introducing conversations about death, explaining:

*“… because we’ve been going through this for a year now, and we’ve had a lot of bad news days. We’ve had good news days, we’ve had blunt conversations, we’ve had sugarcoated conversations. I just find it is easier to go with the preferences I gave you because that’s what has been helpful for me and my child.” *

*(Parent 305)*

Another parent shared that the uncertainty during her child’s initial cancer diagnosis caused significant fear, shaping her current preference for comprehensive, transparent prognostic information: “*This is my second time with this, and as shocking as it is, I don’t want to feel as isolated as I was… It’s just because [before] I didn’t know a lot. I didn’t know [what] I do now. I was living in fear…” (Parent 303)*

### 3.4. Faith

A few patients and some parents described the role of faith in shaping their prognostic communication preferences. For these participants, faith prompted a focus on the present and a reluctance to engage in discussions about the future, grounded in a belief of divine control. As one parent remarked, “*We take it day by day through our faith because that’s just our beliefs and we believe that God is in control. There’s nothing else that we can do.” (Parent 107)*

Additionally, a few parents cited their faith as the reason for rejecting statistical framing, emphasizing ultimate belief in a higher power over medical probabilities. One parent commented, *“So, like if someone, a doctor says there’s only a 20% cure rate… okay you know what the statistics are, but like you’re not God… I’ve seen what I’ve prayed for come to life when I was told that it probably wouldn’t.” (Parent 209)* Parents also discussed relying on faith-based supports within the hospital. Another parent shared, *“We did ask to see a preacher after the fact, and they provided that. Just to answer some questions on why the hell this was happening to our son, and I don’t know.” (Parent 408)*

While some parents embraced faith as the framework through which they navigated their child’s illness and/or a system of support, a few described faith as less relevant in shaping their illness experience, including their communication preferences. As one parent remarked, “*I’m just very different from most people, and I don’t know where it comes from. I’ve been through a lot in my life, and a lot of people thank God. I don’t. I thank myself.” (Parent 110)*

Some oncologists also mentioned faith as a driver of their prognostic communication preferences. A few oncologists explained that, while their own faith did not play a role in their communication practices, it was important to honor the faith-based preferences of patients and families. They discussed how understanding the function of illness-related communication within the context of a family’s spirituality and faith community can help make communication personalized and compassionate:

*“I think religion is critically important and that we acknowledge the belief system of the family… the more I can hear what they want, what their community wants, what their [religious leader] is telling them, the more we can actually be on the same page.” *

*(ON16)*

## 4. Discussion

In this study, pediatric cancer patients, parents, and oncologists reflected on factors that have shaped their prognostic communication preferences. Four distinct themes emerged around personal identity, life experiences, prior medical encounters, and faith as drivers of preferences for prognostic disclosure. Understanding the rationale behind these preferences can help clinicians elicit prognostic communication preferences with greater nuance and specificity, with the goal of better individualizing conversations about prognosis across advanced childhood cancer.

The theme emphasized most across participant cohorts was how individual personality traits played a central role in shaping how they wanted to receive prognostic information. Prior research has shown that when clinicians understand what makes a patient or family unique, it becomes easier to personalize care [22]. In line with this, we advocate for communication skills training to promote the importance of clinicians making a concerted effort to understand the individual personalities and values of patients and caregivers as part of an effective communication approach to elicit prognostic communication preferences and tailor disclosure to align with these preferences.

Notably, a few parents expressed their desire to protect and advocate for their child as driving factors in their communication preferences. These findings build upon previous work on parental identity in serious illness, with many parents reporting that advocating for their child was a core behavior that defined what being a “good parent” meant to them [23]. Additional research has explored how this construct influences the interaction between parents and clinicians, highlighting that clinician acknowledgement of parental roles supports their sense of self-efficacy and satisfaction with decision making [24]. Consistent with these findings, we recommend that clinicians ask parents what being a “good parent” means to them and how the medical team can best support these aspirations. Interestingly, some clinicians expressed a parallel desire to be a “good doctor.” Further research into exploring clinician identity and its impact on communication is warranted to promote effective communication skills training, potentially modeled after the “good parent” framework.

Patients, families, and oncologists often cited general life experience as shaping their prognostic communication preferences. Participants described how formative events and personal growth over time influenced their values, personality traits, and approach to conversations about serious illness. For some, these preferences reflected a lifelong tendency toward realism, emotional expression, or the desire for clarity. Others described how professional roles—including careers in healthcare, military service, or data-driven fields—shaped a need for structure, transparency, or detailed planning in medical discussions. Several oncologists highlighted how their cultural backgrounds shaped their perspectives, particularly noting how expectations around prognosis discussions differ across countries and medical systems. These findings reinforce the importance of considering each individual’s broader life context, including cultural influences and career history, when navigating sensitive prognostic conversations.

Families frequently referenced past medical encounters, both positive and negative, as influential in shaping current communication preferences. These findings echo prior research showing that medical experiences impact how families wish to receive information [25]. Negative past interactions can heighten families’ vigilance and mistrust toward particular communication styles. Clinicians can minimize harm and build trust by asking patients and families about their prior experiences, validating their concerns, and adapting their communication accordingly.

Lastly, faith and spirituality emerged as significant, albeit highly individualized, factors influencing communication practices. Prior analyses have shown that some patients and families want to have the chaplain present during prognostic disclosure, while others perceive spiritual care involvement to be an anxiety-provoking harbinger of impending death [26]. Given the diversity of beliefs and interpretations, clinicians cannot know or understand every spiritual framework. However, it is important for clinicians to develop the skills to ask open-ended questions about how spirituality might influence communication preferences around prognosis and other sensitive topics [27]. This type of nonjudgmental inquiry can preempt stereotyping and foster respectful, individualized care [26].

Given this diversity in values, beliefs, and communication styles, a flexible, patient- and family-centered approach is essential. Clinicians can harness principles of motivational interviewing (MI) and shared decision making (SDM) when exploring patient and family preferences to help align prognostic communication with their values and goals [28]. MI is a communication approach that emphasizes open-ended inquiry, reflective listening, and affirmation of autonomy to support patients and families in expressing their preferences and goals. SDM, meanwhile, focuses on integrating clinical evidence with patient and family values to make collaborative, individualized choices. When applied to prognostic disclosure, these approaches offer a structured yet flexible framework for eliciting what matters most to families, moving beyond a one-size-fits-all model of communication. The goal of high-quality prognostic communication is not simply the conveyance of medical facts but also the creation of a shared understanding about the illness course rooted in the family’s identity, experience, and meaning making. Ultimately, how oncologists talk about prognosis significantly influences how pediatric patients and their parents understand their disease, which in turn influences their ability to make informed decisions that reflect their goals and values. Specific prompts for clinicians to consider that incorporate principles from each of the identified themes are offered in Table 4.

Several limitations for this study are important to note. Most participants were white and female, although purposeful sampling yielded enrollment of Black and biracial participants exceeding institutional demographics. Underrepresentation of Latino participants may be related to the inclusion of English speakers, and future studies extending from this work will purposefully recruit and enroll participants who speak languages other than English. While culture did not emerge as a prominent theme influencing prognostic communication preferences in our findings, prior research has shown that cultural background can significantly shape how patients and families engage in prognostic communication [29]. The discrepancy in these findings may reflect the relative homogeneity of our sample, with most participants identifying within the dominant culture, rather than indicating that cultural factors are unimportant. While many patients and families received care across different sites in addition to the central study site, there may be a selection bias in this study for participants who seek intensive and/or experimental therapies for advanced cancer, which could shape prognostic communication preferences in unknown ways. Further research is needed to understand how factors that influence preferences for prognostic disclosure differ between populations and/or evolve across time.

In summary, this study highlights the complexity of prognostic communication preferences and the importance of understanding families’ perspectives through a broader human-centered lens. By actively exploring the experiences, values, and identities that shape prognostic communication preferences, clinicians can foster trust, clarity, and alignment in some of the most challenging conversations in pediatric oncology. Future research will aim to partner with patients and parents to codesign best practices for eliciting individual communication preferences and the reasons behind them, as well as strategies to honor these preferences in practice.

## Figures and Tables

**Table 1:** Research team attributes and qualifications.

Author	Attributes and Qualifications
E.B.	Female research associate with a bachelor’s degree in Psychology and training in qualitative research methodology.
R.A.K.	Female scientist–practitioner with a PhD in Clinical Psychology and extensive clinical, research, and advocacy experience in psycho-oncology and pediatric palliative care psychology.
C.R.	Female undergraduate student with training in qualitative research methodology.
H.F.	Female research associate with a master’s degree in Anthropology, formal MAXDA training, and expertise in qualitative research methodology.
S.M.	Female research associate with formal MAXQDA training and expertise in qualitative research methodology.
C.C.	Female white pediatric hematology–oncology fellow pursuing graduate-level training in clinical research and qualitative methods
E.C.K.	Female white physician–scientist with a medical degree, a master’s in Public Health, graduate-level training in qualitative research methodology with a focus on communication science, and clinical training and practice in pediatric hematology–oncology and hospice and palliative medicine.
All Authors	No members of the research team had a personal or professional relationship established with study participants prior to study commencement. During the informed consent process, research team members informed participants about the team’s goals/reasons for conducting this research; individual interviewers otherwise did not share personal feelings or reasons for conducting the research with participants during the study process. All study team members share a collective interest in partnering with patients, parents, and clinicians to improve person-centered prognostic disclosure in pediatric cancer.

**Table 2:** Participant demographics.

Participants	*n* (%)
**Parents (*n* = 40)**	
Gender	
Male	8 (20)
Female	32 (80)
Race	
White	24 (60)
Black/African American	14 (35)
Asian	1 (5)
Biracial	1 (5)
Middle Eastern	0 (0)
Ethnicity	
Hispanic/Latino	5 (13)
Non-Hispanic	35 (87)
**Patients (*n* = 25)**	
Gender	
Male	14 (56)
Female	11 (44)
Race	
White	18 (72)
Black/African American	6 (24)
Asian	0 (0)
Biracial	1 (4)
Middle Eastern	0 (0)
Ethnicity	
Hispanic/Latino	3 (12)
Non-Hispanic	22 (88)
Patient age	
12–14 years	13 (52)
15–17 years	7 (28)
18–20 years	3 (12)
21–25 years	2 (8)
**Oncologists (*n* = 20)**	
Gender	
Male	6 (30)
Female	14 (70)
Race	
White	13 (65)
Black/African American	0 (0)
Asian	6 (30)
Biracial	0 (0)
Middle Eastern	1 (5)
Ethnicity	
Hispanic/Latino	1 (5)
Non-Hispanic	19 (95)

Note: Regarding non-participants, 116 eligible participants were approached, of which 23 (19%) refused to participate. Of the 93 participants enrolled, 85 completed interviews.

**Table 3:** Theme frequencies across participant groups.

Theme	Patients *n* (%)	Parents *n* (%)	Oncologists *n* (%)
Personality, Ideals, and Values	13 (52)	22 (55)	9 (45)
General Life Experiences	3 (12)	10 (25)	19 (95)
Previous Interactions with the Medical System	7 (28)	16 (40)	5 (25)
Faith and Spirituality	2 (8)	13 (32.5)	5 (25)

**Table 4:** Questions for clinicians to tailor prognostic communication.

Theme	Example Questions
Faith	“I wonder if you might feel comfortable sharing what gives you hope, meaning, or strength as you face your child’s illness?” “How, if at all, do your faith or spiritual beliefs influence your preferences for talking about prognosis with your child’s care team?” “Is it important to have someone from your faith community (e.g., chaplain, clergy, elder) with you when we review the scan results together next week?” “How can our team honor your spiritual beliefs as we discuss what comes next for [child’s name]?”
Personality, Ideals, and Values	“Different people like to hear information in different ways. Thinking about your own personality, what approach—e.g., big-picture, every detail, or something else—would feel most helpful when we talk about [child’s name]?” “Some families like numbers and statistics, while others prefer to hear more general information about what to expect. What feels right for you?” “What would help you feel better prepared after we talk: written notes, next-step plans, or time to reflect?” “Parents often have a personal sense of what it means to be a ‘good parent’ when their child is sick. What does being a good parent look like for you right now, and how can our team support you?”
General Life Experiences	“Have any past experiences with serious illness—whether yourself, your child, or another loved one—influenced your preferences for hearing information about your child’s illness?” “May I ask what you do for work? People’s work or training can shape how they make sense of medical information. I wonder if your work or training influences the way you’d like us to share news about your child’s illness and care?” “Thinking about challenging situations you’ve faced in the past, what kind of support got you through it?”
Previous Interactions with Medical System	“Tell me about your previous experiences with doctors or hospitals. How has it been for you communicating with medical teams?” “Are there words, phrases, or approaches that have felt unhelpful in past medical conversations?” “Have there been times when information was shared with you or your child in a way that was upsetting or unhelpful?” “What would a good partnership between your family and the care team look like as we move forward?”

## Data Availability

In the context of the rarity of advancing pediatric cancer and the relatively small sample sizes intrinsic to qualitative research, a small risk exists for participant identification even following rigorous de-identification procedures. Given this risk, our research team does not share entire raw data sets upfront to all-comers. We are enthusiastic about sharing de-identified data on a case-by-case basis to researchers under a data-sharing agreement in the setting of an IRB-approved research protocol and explicit assurance that data will be reviewed and analyzed exclusively for research purposes without identification of individual participants.

## Supplementary Materials

The following supporting information can be downloaded at: https://www.mdpi.com/article/10.3390/children12091140/s1, Table S1: COREQ checklist. Reference [14] is cited in the Supplementary Materials.

## Abbreviations

The following abbreviations are used in this manuscript:

RIGHTime	Revealing Information Genuinely and Honestly across Time
COREQ	Consolidated Criteria for Reporting Qualitative Research
MI	Motivational interviewing
SDM	Shared decision making
